# Chinese Herbal Medicine (Zi Shen Qing) for Mild-to-Moderate Systematic Lupus Erythematosus: A Pilot Prospective, Single-Blinded, Randomized Controlled Study

**DOI:** 10.1155/2013/327245

**Published:** 2013-05-08

**Authors:** Linda L. D. Zhong, Zhao Xiang Bian, Jun Hua Gu, Xin Zhou, Yu Tian, Jian Chun Mao, Xiang Jun Chen

**Affiliations:** ^1^Clinical Division, School of Chinese Medicine, Hong Kong Baptist University, Hong Kong; ^2^Department of Rheumatology, Longhua Hospital, Shanghai University of Traditional Chinese Medicine, 725 Wan Ping South Road, Shanghai, China; ^3^Department of Pharmacy, Longhua Hospital, Shanghai University of Traditional Chinese Medicine, 725 Wan Ping South Road, Shanghai, China

## Abstract

*Objective*. The aim of this study is to investigate the effectiveness and safety of a Chinese herbal formula Zi Shen Qing (ZSQ) in the treatment of systematic lupus erythematosus (SLE) in Chinese patients. *Methods*. A randomized controlled trial was conducted over 12 weeks in 84 Chinese patients who reported total scores of SLE Disease Activity Index-2000 (SLEDAI-2000) was from 5 to 14. The primary outcome was the changes of the SLEDAI-2000. The secondary outcomes included score changes of Chinese Medicine Syndromes (CMS), the changes of steroid dosage, the incidence of disease flare-up and biologic markers. *Results*. ZSQ significantly reduced SLEDAI-2000, the total scores of CMS in the treatment group compared with the controlled group (*P* < 0.05). Superiority of ZSQ over controlled group was also observed with greater improvement in the withdrawal dosage of corticosteroids and the incidence of disease flare-up (*P* < 0.05). There were no serious adverse events, and safety indices of whole blood counts, renal and liver functions were normal, both before and after the treatment. *Conclusion*. ZSQ is safe and effective for decreasing SLE disease activity and withdrawal dosage of corticosteroids in the mild to moderate SLE patients with “Deficiency of Qi and Yin” Pattern.

## 1. Introduction

Systematic lupus erythematosus (SLE) is a typical autoimmune disease which affects a variety of organ systems and markedly causes significant physical disfigurement, morbidity, and occasionally mortality [1–3]. Patients with SLE also develop distinct immunologic abnormalities, particularly in the antinuclear, anticytoplasmic, and antiphospholipid antibodies [4–6]. The overall population prevalence is 400 to 100 per 100,000 women, depending on age groups and demographic factors [7]. Although available therapies, such as corticosteroids, hydroxychloroquine, and the new immunosuppressive drugs, have improved the overall survival and severe organ damage, there remains a significant unmet need for quality of life as well as more safe and effective treatments [8–10]. With the unsatisfactory response to current conventional treatments [11], many patients seek help from traditional Chinese medicine (TCM), mostly by taking Chinese herbal medicine (CHM) [12].

According to TCM, SLE can be defined as rheumatism (*Bi Zhen*), edema, and palpitation of heart due to different organic damage and in general divided into excessive and deficient syndromes [13, 14]. A summary of mostly common used Chinese herbal medicines in SLE [15] and the characteristics was listed in Table 1. The former mainly is called heat toxin syndrome and is characterized by the fresh red skin rash, severe joint pain, multiple oral ulcers, high fever, dark colored urine, crimson tongue with yellow coating and rapid sunken pulse [16]. The latter has manifestations as deficiency of Qi and Yin with benign skin rash, minor joint pain, lack of strength, shortness of breath, dry throat and mouth, vexing thirst, small amount of urine, red tongue with dry and/or few coating, and rapid string-like pulse or rapid fine pulse [17]. Therapeutic principles and practices vary accordingly. The formula Zi Shen Qing (ZSQ) was originated from classic CM formulae Yu Ping Feng San and comprised of six herbs (Table 2). Through the combination of these herbs, ZSQ can tonify Qi, enrich Yin, remove internal heat, and clear the toxin [18]. 

Our research team has studied ZSQ for SLE from 1990s. We found that ZSQ could normalize the low C3 and C4 as well as reverse anti-dsDNA positivity [19]. It also provided reduction of corticosteroid dosage and prevention of severe disease flare [20, 21]. Previous studies had many limitations, for example, proper randomization procedures were absent, blinding was inadequate, strict eligible criteria (including Western Medicine and Chinese Medicine Syndrome) were not clearly defined, quality control was not conducted, and so forth [17–21]. To further assess the effectiveness and safety of ZSQ in the mild-to-moderate SLE with “deficiency of Qi and Yin” pattern, well-controlled granules and standard clinical parameters were performed in this pilot clinical trial.

## 2. Methods

### 2.1. Participants and Setting

All the participants were recruited via advertisements in local newspapers and outpatient specialist clinics of rheumatology in LongHua Hospital, Shanghai, China, from 1st of March 2006 to 31st of January 2009 for preliminary screening. Preliminary telephone interviews were conducted to determine whether potential participants met the broad inclusion criteria. Participants were then screened by a rheumatologist to ensure their medical suitability before entering into the trial. Diagnosis of SLE was based on 1997 American College of Rheumatology (ACR) classification criteria for SLE [22]. Diagnosis of “deficiency of Qi and Yin” SLE was based on the criteria described in *Guidance for Clinical Research of Chinese Herbal Medicine 2002* and *Therapeutic Effect of Disease and Syndromes in Traditional Chinese Medicine *[23, 24]. All participants presented with any three of the chief symptoms and any two of the simultaneous symptoms had diagnosis of “deficiency of Qi and Yin”. Chief symptoms included (i) red skin rashes; (ii) dry mouth and oral ulcers; (iii) muscle or joint pain; (iv) vexing heat in the body; (v) red tongue with dry and/or few coating; (vi) rapid string-like pulse or rapid fine pulse. Simultaneous symptoms included (i) low back pain and lack of strength; (ii) dry mouth and eyes; (iii) tinnitus; (vi) hair loss. 

Participants were recruited only from those who (i) age 12–65 years old; (ii) having diagnosis of SLE; (iii) have total scores of SLEDAI-2000 from 5 to 14 indicating mild-to-moderate disease activity; (vi) have diagnosis of “Deficiency of Qi and Yin” CM syndrome; (v) have stable treatment regimen for at least 30 days with prednisone alone (5–30 mg/day) or combined with nonsteroidal anti-inflammatory drugs; (vi) have seropositivity as defined by 2 positive ANA or anti-dsDNA test results (ANA titer ≧ 1 : 80 and/or anti-dsDNA antibody level ≧ 30 IU/mL), of which ≧1 test result had to be obtained during screening. Exclusion criteria included serious intercurrent illness, severe active lupus nephritis, severe central nervous system manifestations, CHM allergies, severe psychiatric disease, and pregnancy. The study was conducted in accordance with the Declaration of Helsinki and was approved by the Institutional Review Board of Shanghai University of Traditional Chinese Medicine/LongHua Hospital. All participants gave their written, informed consent and were free to withdraw at any time from the trial. 

### 2.2. Study Design

The study consisted of a 2-week baseline period and 12-week treatment period. Eligibility screening was performed, including questionnaires of SLE CM syndrome scores; SLEDAI-2000 and information on demographic characteristics, medications, smoking, and alcohol consumption were also collected. After the eligible screening visit, participants entered a 2-week run-in phase to obtain baseline data. These consisted of SLEDAI scores, whole blood count, liver and renal function tests, immunologic index as C3, C4, IgG, ANA, anti-dsDNA, NK, and sIL-2R. Two blood samples were obtained at baseline and endpoint by our research nurse and checked by the Medical Laboratory, LongHua Hospital, Shanghai University of TCM. These data were used to ensure the minimum eligibility criteria for each participant and to screen out respondents with potential poor compliance. After the run-in phase, patients who met entry criteria were randomly assigned to either the ZSQ group or the controlled group. Hydroxychloroquine (HCQ) sulfate tablet (Shanghai Zhongxi Pharmaceutical Co., Ltd.) was used as the controlled medication, which was prescribed as 100 mg twice a day. We have a standard plan for glucocorticoids (GC) according to Kelley's Textbook of rheumatology, 8th edition [6]. Low-dose GC therapy (≤7.5 mg prednisone equivalent per day) was generally used to control mild SLE activity. In moderate SLE, GCs were used as either single or background therapy in combination with immunosuppressive agents, at doses of 0.5 to 1 mg/kg prednisone equivalent in a single dose usually in the morning. When combined with immunosuppressive agents, the GC dose should rarely exceed 0.5 to 0.6 mg/kg prednisone due to concerns for infections and other toxicities. Tapering of GC dose started after the first 4 to 6 weeks of therapy, targeting a dose of 0.25 mg/kg every other day at 2 to 3 months, which was acceptable for long-term use. 

Consultations were conducted every 4 weeks over the 12-week treatment period. To maintain blinding, the investigators were not allowed to ask the participants about the types of the interventions, and the participants were instructed not to reveal their interventions to the investigators and discuss, related issues with them. Every participant was given a 4-week supply of medication at each treatment visit by the pharmacists who had no connection to the research. In addition, responses to the SLEDAI and CM syndrome scores were assessed by independent assessor who was also blinded to patients' interventions. Compliance was controlled by counting the remaining sachets. A participant was considered compliant if she/he consumed not less than 80% of the daily dose of the study medication.

### 2.3. Interventions

#### 2.3.1. CHM Intervention

ZSQ is composed of Radix astragali, *Rehmannia glutinosa libosch*, Fructus Corni, *Paeonia lactiflora*, Herba Hedyotis Diffusae, and Cortex Moutan Radicis. The composition and action of each herb is summarized in Table 2. Patients are instructed to dissolve a sachet of granules (10 g) in 200 mL of hot water; they take this solution orally twice daily for 12 weeks. The preparation of herbs and methods of quality control had been published in our previous paper [25, 26] and listed in Supplement 1 (see supplementary material available at http://dx.doi.org/10.1155/2013/327245) About one gram of three batches granula ZSQ (Lot. 060611, 060612, 060613) was, respectively, extracted by about 9 mL methanol for 20 min under ultrasound, and then methanol was added till the total volume was 10 mL. After filtration, supernatant was centrifuged at 10,000 r/min for 10 min and filtered through 0.22 *μ*m PTFE syringe filter. Filtrate was diluted 10 times with methanol, and an aliquot of dilution (20 *μ*L) was injected into HPLC-DAD analysis.

#### 2.3.2. WM Intervention

Fen Le tablet is selected as the active control, for which each tablet consists of 100 mg hydroxychloroquine sulfate. It was manufactured by Shanghai Zhongxi Pharmaceutical Co., Ltd (http://www.shzxyy.com/English/index.asp). Patients were instructed to take 1 tablet twice daily for 12 weeks. 

All ZSQ granules were prepared by Xiu Long Pharmaceutical (Shanghai.) Co. Limited. The entire manufacturing process, from authenticating the raw materials to the final products, is in strict compliance with the standards of Good Manufactory Practice (GMP) and Chinese Pharmacopoeia [15]. Acute toxicity test of ZSQ granules on mice had been done for monitoring the toxicity of this formula. The results showed that maximum tolerance dosage was 45 g/kg, equal to 150 times of the dose tested. The chemical compositions of the final products are analyzed, while contamination with heavy metals, toxic elements, microbes, and pesticide residue was tested by an SGS laboratory. The ZSQ granules were packed in sealed opaque aluminium sachets and put in zip lock bag (28 sachets each), while WM intervention was put in a plastic bottle (56 tablets each). 

### 2.4. Outcome Measures

The primary outcome was the changes of the SLEDAI-2000 [27]. SLEDAI was originally developed and validated as a clinical index for the measurement of disease activity in SLE and has been used as a global measurement of disease activity in SLE since 1985 and modified by a panel of experienced rheumatologists with expertise in SLE, using well-established group techniques and index development methodology in 2000. The index has been used by both researchers and clinicians and also is shown to be valuable as a predictor for mortality [28–30]. 

The secondary outcomes included score changes of Chinese medicine syndromes (CMSs), the changes of steroid dosage, and the incidence of lupus flares. The items of CMS and description of rating were listed in Table 3. Mild-to-moderate flares were defined as one or more of the following features: (1) a >3-point change in the SLEDAI score, with a total score of ≤12; (2) new or worsening discoid, photosensitivity, or other rash attributable to lupus, nasopharyngeal ulcers, pleuritis, pericarditis, arthritis, or fever not attributable to infection; (3) an increase in the prednisone dosage, but not to >0.5 mg/kg of body weight per day. Severe flares were defined as (1) an SLEDAI score of >12; (2) new or worsening central nervous system (CNS) involvement, vasculitis, glomerulonephritis, myositis, thrombocytopenia (platelet count <60 × 10^9^ cells/liter), or hemolytic anemia (hemoglobin level <70 gm/liter or a decrease in the hemoglobin level of >30 gm/liter over a 2-week period), each of which required doubling of the corticosteroid dosage to a final dosage of >0.5 mg/kg/day or hospitalization; (3) any manifestation requiring an increase in the dosage of prednisone or to >0.5 mg/kg/day [31]. Assessed biologic markers included serums IgG, C3, C4, anti-dsDNA, ANA, NK, and sIL-2R. Serologic assessments were conducted using enzyme-linked immunosorbent assay, except for ANA. 

### 2.5. Randomization and Blinding

A random sequence was prepared by staff at the GCP center of Longhua Hospital, with no connection to the study. Block randomization was carried out in a 1 : 1 ratio according to the sequence generated with Random Allocation Software (version 1.0.0), Isfahan, Iran. Randomization codes were kept in sealed, opaque envelopes labeled with consecutive random numbers and kept in a location far away from the clinical setting where participants were assessed. Emergency envelopes with the randomization codes were kept by principle investigator. Treatment assignments were not revealed to all the investigators (including outcome assessor), until the study was completed. The investigators, nurses, all other participating medical staff, and the assessors remained unaware of intervention assignments throughout the trial. Only the participants and pharmacists knew the intervention and were instructed not to tell the investigators/assessors about the intervention. Assessment of the success of blinding to the investigators was conducted at the end of the trial.

### 2.6. Sample Size Calculation

The sample size calculation was based on the primary outcome measure. Previous studies of hydroxychloroquine (HCQ) sulfate tablet in the reduction of SLEDAI were about 30% [32]. Our pilot observational study findings were consistent with a reported double-blinded clinical study of CHM on SLE, in that at least 15% reduction in SLEDAI was reported in the CHM group compared to the control [33]. We assumed a difference of at least 15% between ZSQ and HCQ. The sample size in each group was determined by using the formula derived by Whitley and Ball [34]:

(1) $$\begin{array}{c} n=\frac{\left[p_{1}\left(1-p_{1}\right)+p_{2}\left(1-p_{2}\right)\right]}{{\left(p_{1}-p_{2}\right)}^{2}}\times C_{p,\text{power}}. \end{array}$$

When type I: *α* = 0.05, *β* = 0.20; power (1 − *β*) = 80%; *p*_1_ = 30%, *p*_2_ = 45% and when two-tailed level *α* and power were set at 0.05 and 80%, respectively. 35 participants were needed in each group. Estimating about 20% dropouts over 8 weeks of treatment, the number of each group was 42, and a total of 84 participants were needed for this study. 

### 2.7. Statistical Analysis

All efficacy and safety analyses were conducted according to the intention-to-treat (ITT) principle. Missing values were imputed by the last-observation-carried-forward method. The statistical analysis was performed using the Statistical Packages of Social Sciences (SPSS) for Windows version 16.0. The statistical significance was defined as two-sided *P* value of <0.05. Baseline characteristics were reported as mean (SD), frequency, and percentages. Baseline differences between the groups were assessed with the use of Student's *t*-test for normally distributed continuous variables and nonparametric Mann-Whitney *U* test for nonnormally distributed. For categorical variables, chi-squared test or Fisher's exact test were used. Comparisons between groups were conducted by using an analysis of covariance (ANCOVA) with baseline as covariate. All items of CMS were compared between treatment groups for each 4 week using ANCOVA, with treatment group as a factor in the model and baseline as the covariate. The change from baseline to 12 weeks in score of SLEDAI and CMS was tested with repeated measure analysis of variance (ANOVA). Within-group differences were assessed with paired *t*-test for normally distributed data and Wilcoxon signed-rank test for nonnormally distributed data. For biological markers and safety indices, ANCOVA was used to compare difference between two groups, adjusting for baseline covariates.

## 3. Results

### 3.1. Participant Flow

**Figure 1:**
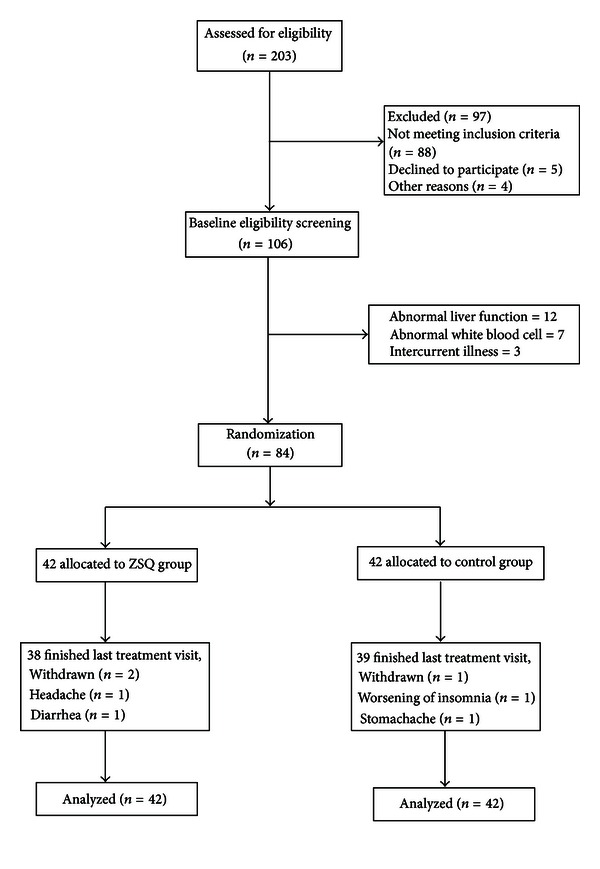
Participant flow diagram.

The participant flow was listed in Figure 1. In total, 203 Chinese SLE patients were recruited from 1st of March 2006 to 31st of January 2009 for preliminary screening. 106 participated in baseline eligibility screening for randomization. 12 participants were screened out due to abnormal liver function, 7 participants were screened out due to abnormal white blood cell numbers, and 3 were screened out due to intercurrent illness. A total of 7 participants (4 from the ZSQ group and 3 from the control group) withdrew during the study period; reasons were listed in the flow diagram. 

### 3.2. Baseline Data

The baseline characteristics of the participants were summarized in Table 4. The mean age of ZSQ group was 32.7 ± 13.6 years, and the mean age of control group was 34.8 ± 14.3 years (*P* = 0.46). The mean prednisone dosage of ZSQ group was 10.2 ± 6.4 mg/d, and control group was 10.6 ± 6.6, respectively (*P* = 0.62). The disease duration was 4.7 ± 4.4 months in ZSQ group and 5.0 ± 4.6 months in control group (*P* = 0.79). The baseline characteristics of the two groups were balanced (*P* > 0.05).

### 3.3. Primary Outcome Assessment

The total scores of SLEDAI decreased from 10.5 ± 2.2 to 5.1 ± 1.5 in the ZSQ group and from 10.6 ± 2.1 to 7.0 ± 1.9 in the control group, during the 12-week treatment period (*P* = 0.03; Table 5).

### 3.4. Secondary Outcome Assessment

The total scores of CMS decreased from 19.5 ± 3.9 to 12.3 ± 4.3 in the ZSQ group and from 19.7 ± 3.9 to 14.6 ± 5.2 in the control group, during the 12-week treatment period (*P* = 0.04; Table 5). The mean prednisone dosage decreased from 10.2 ± 6.4 to 3.6 ± 3.1 in the ZSQ group and from 10.6 ± 6.6 to 6.6 ± 5.6 in the control group during the 12-week treatment period (*P* = 0.01; Table 5). The incidence of mild-to-moderate flare-up was 11.9% in the ZSQ group and 19.0% in the control group after the 12-week treatment period based on SLE Flare Index (*P* = 0.02; Table 5), and there was no incidence of severe flare-up in both of the groups. 

### 3.5. Biologic Markers

ZSQ treatment produced sustained reductions in anti-dsDNA and IgG level as well as increased C3 and C4 concentrations, compared with control group (*P* < 0.05, Table 5). The NK cell activity was increased from (14.5 ± 8.8)% to (16.7 ± 9.1)% in ZSQ group and from (14.0 ± 6.2)% to (16.3 ± 4.2)% as well as increased the level of sIL-2R from (40.8 ± 8.6) pM to (37.2 ± 12.5) pM in ZSQ group and from (40.1 ± 14.8) pM to (38.6 ± 13.8) pM in control group, but there is no significance between the two groups (*P* > 0.05). 

### 3.6. Safety and Adverse Events

Whole blood count, renal and liver functions, urine test, and EKG were checked at baseline and after treatments. There were no differences between the two groups in these safety indices. During the study, very few patients experienced slightly adverse events including headache, dry skin, diarrhea, insomnia, and irregular menstrual period and so forth (Table 6). 

### 3.7. Assessment of Blinding and Compliance

Success of blinding was evaluated by asking the investigators which treatment they thought patients had received at each consultation. Overall the investigators identified 45.2% correctly of their treatment (38 among 84 patients), whether ZSQ or control medications.

Compliance at 12-week treatment was fair with three withdrawn in control group and four withdrawn in ZSQ group (Figure 1). The overall compliance with medication in the remaining participants was good. 92% of participants were found to have taken at least 80% of scheduled doses, as determined by counting remaining medication sachets.

## 4. Discussion

In this pilot prospective, single-blinded, and randomized controlled study, we found that ZSQ was superior over controlled group in decreasing the total scores of SLEDAI-2000 and CMS, and effectively reducing steroid dosage as well as the frequency of disease flare-up. In the specific domains of “deficiency of Qi and Yin,” ZSQ can also relieve the symptoms of dry mouth, hair loss, and muscle or joint pain. Moreover, the sustained benefits of ZSQ were observed in the follow-up period. 

The choice of the positive control not the placebo control is mainly based on the ethical consideration. Large scales of clinical studies had shown that SLE was mostly like flare when using low dose of corticosteroids alone [35]. Antimalarial drugs, mainly chloroquine (CQ) and HCQ, are commonly prescribed to SLE patients with skin and joint manifestations and are increasingly identified as an adjuvant treatment for achieving remission in severe lupus [36, 37]. Recent studies also indicated that it could prevent irreversible organ damage [38]. The adverse effects of antimalarial drugs included retinopathy, myopathy, and minor myocardiopathy and so forth. In addition, these drugs had antiplatelet and hypolipidemic effects. In the setting of systemic autoimmune diseases, like SLE, antimalarial drugs act upon a panoply of targets without increasing risk of opportunistic infections.

ZSQ comprised of Radix Astragali,* Rehmannia glutinosa Libosch*, Fructus Corni, Rhizoma Atractylodis Macrocephalae, Herba Hedyotis Diffusae, and Cortex Moutan Radicis. It originates from the classic formula “Yu Ping Feng San (YPFS),” and it has been commonly used to regulate the immune systems [39]. In the study of healthy volunteers, CD4/CD8 ratio of T-lymphocytes was significantly increased after taking the YPFS regimen for 14 days, and no such increase was observed after the consumption was stopped (day 29) as well as during the entire control study period [40]. Our animal study also indicated that three different dosages of ZSQ granules (low dosage of 1.6 g/kg/day, moderate dosage of 3.2 g/kg/day, high dosage of 6.4 g/kg/day) would increase the serum level of total white blood cells, and only moderate and high dosage groups would increase the levels of CD3, CD8, and CD4 [41]. In our study, we also found that the concentration level of C3 and C4 was increased, which plays a central role in the activation of complement system. Complement activation is an efficient means to coat antigens so that they adhere to peripheral blood cells and are then phagocytosed or carried to the spleen and liver for disposal [41, 42]. In these organs, the immune complexes are transferred to tissue macrophages for destruction or an immune response. Complement measurements are helpful in diagnosing and following patients with systemic lupus erythematosus. Low C4 and C3 in the presence of anti-double-stranded antibodies have nearly a 100% specificity for lupus [43].

The beneficial roles of NK cells are involved in immune tolerance and can ameliorate or prevent tissue inflammation. In SLE patients, the reduced proportion and function of NK cells correlate with disease activity, and NK cells correlate inversely with IgG levels [44]. In current study, we found that the results were consistent with previous study, and ZSQ increased the NK cell activity and decreased the IgG level compared with control group [45]. In SLE patients, it has been found that activated T cells and B cells release both interleukin-2 and a soluble form of interleukin-2 receptor (sIL-2R) [46]. The serum IL-2R level thus has been used as a marker for disease activity [47]. In this study, we found that the level of sIL-2R in ZSQ group and in control group increased after treatment, but there is no significance between the two groups.

The corticosteroids exert broad inhibitory effects on immune responses mediated by T and B cells, and their rapid onset of actions made them widely used in managing acute SLE manifestations [48–50]. However, the adverse effects involve early (mood effects, acne, myalgias, and infections); later (metabolic); late-onset (osteoporosis, avascular bone necrosis, cataract, and cardiovascular disease) events [51]. Furthermore, about 30% patients responded relapse when steroids are withdrawn and become steroid dependent [52]. So reduction to the dose of corticosteroid and induction of the remission of SLE are of paramount importance. ZSQ was investigated in combination with corticosteroids in this pilot study, and it was superior over controlled group in reducing the dosage of corticosteroids. The incidence of lupus flares in ZSQ group was 11.9% and in control group was 19.0%, respectively, which may indicate the preventive effects of SLE flare-ups in ZSQ group.

Our study has several limitations. First, it was a single-blinded clinical trial without a placebo group, and it may affect the patients' response to the assessments. Although a double-blinded, placebo-controlled trial would have been optimal, we believe that the investigators were effectively blinded because the patients were instructed not to reveal the interventions and the assessment of blinding was successful. Second, self-selection bias is likely present, as most of the study subjects were recruited through newspaper advertisements and agreed to participate in this clinical trial. Most of them expressed negative views of immunosuppressive agents and corticosteroids, during intake interviews, and many may even have had high prior expectations regarding the effectiveness of Chinese medicine. Such expectations may have led to inaccuracies in reporting symptoms. Third, our study was a small, single center trial in a single ethnic Chinese population, and thus, findings may not be broadly generalizable. Although our findings indicated that ZSQ was superior to HCQ in some outcome measurements, the tentatively positive effects shown in the present study should be interpreted with caution. 

In conclusion, our study shows that the Chinese herbal medicine ZSQ is effective and in the mild-to-moderate Chinese SLE patients with “deficiency of Qi and Yin” pattern in reducing SLE disease activity and withdrawal dosage of corticosteroids. It is safe and well tolerated, with no serious adverse events noted during the study period. Larger-scales, multicenter, active controlled and/or placebo-controlled, clinical studies of ZSQ on more diverse populations are needed to further confirm these findings. 

## Supplementary Material

About one gram of three batches granula ZSQ (Lot.060611,060612, 060613) were respectively extracted by about 9 ml methanol for 20 min under ultrasound, then methanol was added till the total volume was 10 ml. After filtration, supernatant was centrifuged at 10,000 r/min for 10 min, and filtered through 0.22 *μ*m PTFE syringe filter. Filtrate was diluted 10 times with methanol and an aliquot of dilution (20 *μ*l) was injected into HPLC-DAD analysis. The details of the quality control was listed as below.

## Figures and Tables

**Table 1:** Comparison of different Chinese herbal medicines in SLE.

Chinese name	Full scientific name	Action in TCM	Immunomodulatory effects
Ren Shen	Radix Ginseng	Replenishes *qi* and strengthen *yang* Tonify the spleen and lungs	Stimulates the bone marrow and improves the phagocytic function of monocytes
Huang Qi	Radix Astragali	Tonify spleen and lung* qi*, and *wei qi *	Enhancement of T-cell proliferation, promoting NK cell killing effects
Gou Qi	Wolfberry Fruit	Nourishes liver and kidney and tonifies *yin *	Activates the thymus cell proliferation and improves IL-2 and white blood cells
He Shou Wu	Radix Polygoni Multiflori	Nourishes kidney and blood	Enhancement of C3b receptor, promoting interferons (IFNs) production
Ling Zhi	Ganoderma Lucidum	Boosts heart* qi* and calms *shen *	Inhibits platelet aggregation and improves CD^4+^ level and CD^4+^/CD^8+^ level
Gan Cao	*Glycyrrhiza Uralensis *	Tonifies *qi* and clears heat and toxic fire	Modulates serum level of IgE, IgA, and IgM antibodies, decreases serum level TNF-*α*, and induces B cell maturation

**Table 2:** Composition and function of ZSQ granules.

Chinese name	Full scientific name	Parts of plant use	%	Dose of dry plant	Constituent	Dose after extraction	Function in Chinese medicine
*Huang Qi *	*Radix Astragali *	Dry root		15	Astragaloside	3.75	Tonifies Qi
*Sheng Di *	*Rehmannia glutinosa *Libosch	Dry root		15		3.75	Nourishes Yin
*Shan Zhu Yu *	Fructus Cornus	Dry fruit		5	Loganin	1.25	Tonifies the liver and kidney and retains the essence
*Bai Shao *	*Paeonia lactiflora *	Dry root		15	Paeoniflorin	3.75	Nourishes blood and preserves Yin
*Dan Pi *	Cortex Moutan Radicis	Dry root and stem		15	Paeonol	3.75	Clears deficient fire and heat
*She She Cao *	Herba Hedyotidis Diffusae	Dry leaf		15	Oleanic acid	3.75	Clears heat and resolves toxins

Total			100	80		20	

**Table 3:** Chinese medicine syndromes rating scales.

Item	Not at all 0	A little 2	Quite a bit 4	Extremely 6	Scores 0–6
Red skin rash with flat or raised erythema					
Dry mouth and oral ulcers					
Muscle or joint pain involving 2 or more joints					
Vexing heat in the body with or without fever					
Low back pain and lacking in strength					
Dry throat and eyes					
Intermittent or persistent tinnitus					
Hair loss					

Total scores					

**Table 4:** Baseline demographic and clinical characteristics of the patients.

Characteristic	ZSQ group (*n* = 42)	Control group (*n* = 42)	*P* value
Age, yr	32.7 ± 13.6	34.8 ± 14.3	0.46
Education, yr	10.5 ± 3.7	10.6 ± 3.9	0.35
BMI, kg/m^2^	22.1 ± 2.6	22.7 ± 3.1	0.44
Female (*n*, %)	39 (92.8%)	38 (90.48%)	0.67
Disease duration, yr	4.7 ± 4.4	5.0 ± 4.6	0.79
Dosage of prednisone, mg/d	10.2 ± 6.4	10.6 ± 6.6	0.62
SLEDAI scores	10.5 ± 2.2	10.7 ± 2.1	0.51
SLE manifestations (*n*, %)			
Nephritis	32 (76.2%)	34 (81.0%)	
Arthritis	20 (47.6%)	18 (42.9%)	
Dermatitis	24 (57.1%)	21 (50.0%)	>0.05
Central nervous system	2 (4.8%)	1 (2.4%)	
Lymphopenia	9 (21.4%)	8 (19.0%)	
Thrombocytopenia	6 (14.3%)	7 (16.7%)	
Pericarditis	1 (2.3%)	0 (0.0%)	
Total scores of CMS	19.5 ± 3.9	19.7 ± 3.9	0.80
Anti-dsDNA (IU/mL)	89.8 ± 72.9	89.2 ± 80.5	0.63
C_3_ (g/L)	0.8 ± 0.3	0.8 ± 0.2	0.47
C_4 _(g/L)	0.2 ± 0.1	0.3 ± 0.1	0.61
IgG (g/L)	18.7 ± 5.4	17.9 ± 2.8	0.38
NK cell activity (%)	14.5 ± 8.8	14.0 ± 6.2	0.78
sIL-2R (pg/mL, PM)	40.8 ± 8.6	40.1 ± 14.8	0.80

**Table 5:** Comparison of the main outcome measures within and between ZSQ and control groups.

Outcome measures	ZSQ (*n* = 42)			Control (*n* = 42)			
	Mean	SD	Within-group *P* value^a^	Mean	SD	Within-group *P* value^a^	Between-group *P* value^b^
SLEDAI scores							
Baseline	10.5	2.2		10.6	2.1		0.35
Endpoint (12-week)	5.1	1.5	<0.01	7.0	1.9	<0.01	0.03
CMS scores							
Baseline	19.5	3.9		19.7	3.9		0.80
Endpoint (12-week)	12.3	4.3	<0.01	14.6	5.2	<0.01	0.04
Mean prednisone dosage							
Baseline	10.2	6.4		11.0	6.6		0.39
Endpoint (12-week)	3.6	3.1	<0.01	6.6	5.6	<0.01	0.01
Mild-to-moderate flare-up at endpoint (*n*, %)	5 (11.9)			8 (19.0)			0.02
Severe flare-up	0 (0.0)			0 (0.0)			
Anti-dsDNA (IU/mL)							
Baseline	89.8	72.9		89.2	80.5		0.63
Endpoint (12-week)	76.3	61.6	<0.01	83.4	72.6	0.08	<0.01
C_3_ (g/L)							
Baseline	0.8	0.3		0.8	0.2		0.47
Endpoint (12-week)	1.2	0.3	0.02	0.8	0.3	0.27	0.02
C_4_ (g/L)							
Baseline	0.2	0.1		0.3	0.1		0.61
Endpoint (12-week)	0.3	0.2	0.04	0.2	0.1	0.27	0.04
IgG (g/L)							
Baseline	18.7	5.4		17.9	2.8		0.38
Endpoint (12-week)	17.0	5.0	<0.01	17.8	3.2	0.66	0.03
NK cell activity (%)							
Baseline	14.5	8.8		14.0	6.2		0.78
Endpoint (12-week)	16.7	9.1	<0.01	16.3	4.2	<0.01	0.29
sIL-2R (pM)							
Baseline	40.8	8.6		40.1	14.8		0.80
Endpoint (12-week)	37.2	12.5	<0.01	38.6	13.8	<0.01	0.11

^a^Results from paired *t*-test to examine the effects of within-group, baseline versus 12-week, and 12-week versus 3-month followup.

^b^Results from an analysis of covariance (ANCOVA) with baseline as covariate, ZSQ group versus control group.

**Table 6:** Summary of adverse events.

Items	ZSQ group (*n* = 42), no. (%)	Control group (*n* = 42) no. (%)^a^
Headache	6 (14.3)	4 (9.5)
Nausea	2 (4.8)	1 (2.4)
Diarrhea	6 (14.3)	4 (9.5)
Insomnia	5 (11.9)	2 (4.8)
Dry skin	5 (11.9)	8 (19.0)
Epigastric pain	3 (7.1)	3 (7.1)

^a^ *P* value was calculated from Fisher's exact test and *P* > 0.05.
