# Supplementary material for: Chinese Herbal Medicine (Zi Shen Qing) for Mild-to-Moderate Systematic Lupus Erythematosus: A Pilot Prospective, Single-Blinded, Randomized Controlled Study
Source: Evid Based Complement Alternat Med. 2013 May 8;2013:327245. doi: 10.1155/2013/327245 (PMC3662182; doi:10.1155/2013/327245)
Supplement: Supplementary file 1 — About one gram of three batches granula ZSQ (Lot.060611,060612, 060613) were respectively extracted by about 9 ml methanol for 20 min under ultrasound, then methanol was added till the total volume was 10 ml. After filtration, supernatant was centrifuged at 10,000 r/min for 10 min, and filtered through 0.22 μm PTFE syringe filter. Filtrate was diluted 10 times with methanol and an aliquot of dilution (20 μl) was injected into HPLC-DAD analysis. The details of the quality control was listed as below. [file 327245.f1.pdf]

Supplement 1: Quality control of ZSQ

Equipment

An Agilent 1100 HPLC system (Agilent technology, CA, USA) consisted of a degasser, an autosampler, a column thermostat, a quaternary pump and an Diode Array Detector (DAD);

Materials and chemicals

Standards of loganin and paeoniflorin (98%) were purchased from the National Institute for the Control of Pharmaceutical & Biological Products (Beijing, China). HPLC-grade acetonitrile and methanol were obtained from DIMA Technology Inc (USA). Doubled deionized water was used for all aqueous solutions and mobile phases. All other reagents were standard laboratory reagents of analytical grade. The crude drugs were collected from the cultivation base in the different regions of china and were air-dried according to the procedure described in the Chinese Pharmacopoeia (2010 edition). All were identified by prof. Xiujia Zhou (professor of Shanghai university of traditional Chinese medcine). The granula were prepared by Xiu Long pharmaceutical company.

Determination of paeoniflorin

HPLC analysis for paeoniflorin was performed on an Agilent reversed-phase C18 column (4.6×250mm, 5 μm) coupled with C18 guard column. The mobile phase was consisted of 17% acetonitrile and 83% phosphate aqueous (0.1%) solution at the flow rate of 1ml/min. Detective wavelength was 230nm. Column temperature was 40℃

Determination of loganin

HPLC analysis for loganin was carried on a Chromasil reversed-phase C18 column (4.6×250mm, 5 μm) with C18 guard column. The mobile phase was 9.2% acetonitrile aqueous solution at the flow rate of 1ml/min. Detective wavelength was 240nm. Column temperature was room temperature.

Sample preparation

About one gram of three batches granula ZSQ (Lot.060611,060612,060613) were respectively extracted by about 9 ml methanol for 20 min under ultrasound, then methanol was added till the total volume was 10 ml. After filtration, supernatant was centrifuged at 10,000r/min for 10min, and filtered through 0.22 μm PTFE syringe filter. Filtrate was diluted 10 times with methanol and an aliquot of dilution (20μl) was injected into HPLC-DAD analysis.

Statistic

Statistical differences were evaluated using ANOVA when appropriate.

Results

Under developed methods, paeoniflorin and loganin were well separated (Figure 1,3). There was no disturbance at the same retention time in the negative sample (Figure 2,4).

Results showed (Table 1): contents of paeoniflorin and loganin in three batches of granula ZSQ were respectively in the range of 4.81 ~ 5.07 $mg \cdot g^{-1}$ and 0.35~0.37 $mg \cdot g^{-1}$consistent. Relative standard Deviations (RSDs) of three batches were below 2%, indicating contents of different batches consistent. This proved that our preparation process of granula ZSQ was stable as well as quality of granula ZSQ was controlled and stable, which was suitable for future clinical research.

Table 1 Determination of paeoniflorin and loganin in granula ZSQ (n=3)

Lot No.	Paeoniflorin		loganin	
	content ($mg \cdot g^{-1}$)	RSD(%)	content ($mg \cdot g^{-1}$)	RSD (%)
060611	5.07±0.0427	0.84	0.35±0.0062	1.81
060612	4.81±0.0490	1.02	0.35±0.0048	1.37
060613	4.90±0.0216	0.44	0.37±0.0036	0.98

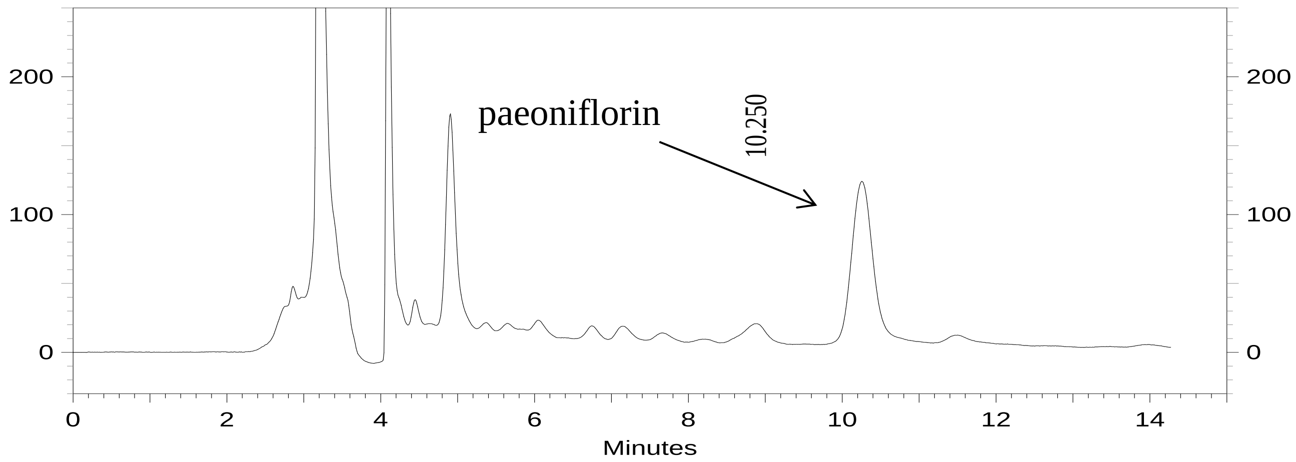

Figure 1 Determination of paeoniflorin for granula ZSQ

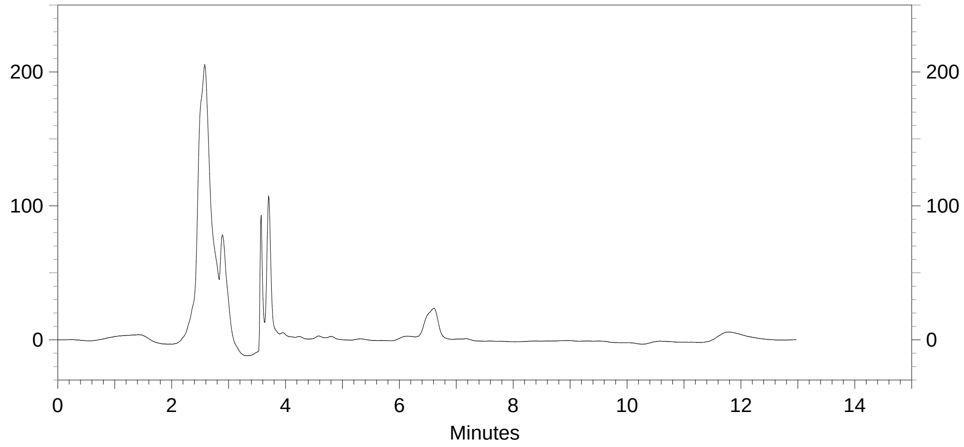

Figure 2 chromatogram of granula ZSQ without Radix paeoniae rubra

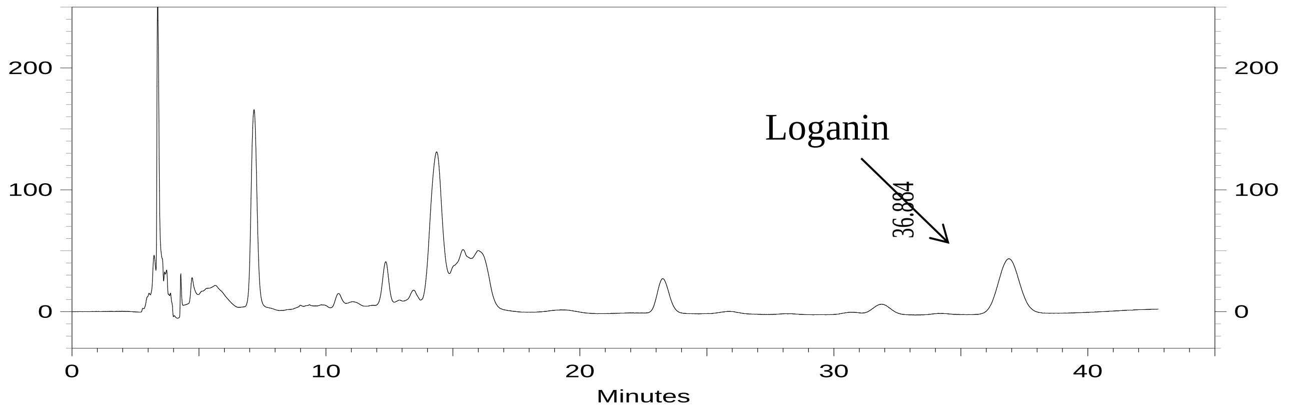

Figure 3 Determination of loganin for granula ZSQ

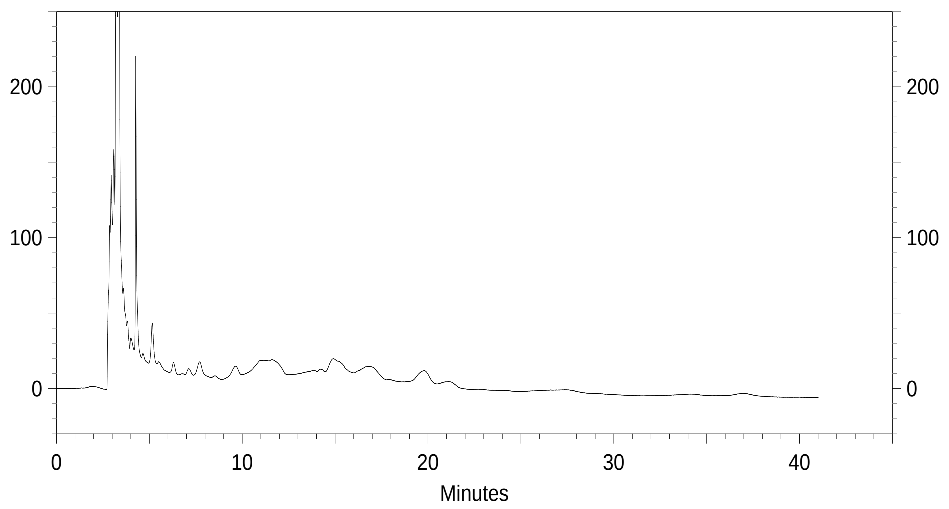

Figure 4 chromatogram of granula ZSQ without Fructus corni,
